# Lack of association of hepatitis C virus (HCV) antibodies and juvenile onset systemic lupus erythematosus (jSLE)

**DOI:** 10.1186/1546-0096-9-S1-P268

**Published:** 2011-09-14

**Authors:** Cláudia Goldenstein-Schainberg, Ana P Nascimento, André LS Hayata, Eloisa Bonfá

**Affiliations:** 1 Rheumatology Division, Faculdade de Medicina da Universidade de São Paulo, São Paulo, SP, Brazil

## Background

The association between viral infections such as hepatitis C virus (HCV) and autoimmunity has been proposed. Antibodies against HCV have been demonstrated in sera from up to 13% adult patients with SLE, however data regarding juvenile onset SLE (jSLE) is lacking despite potential increased risk factors associated.

## Aim

To determine the prevalence and possible association of HCV infection and jSLE.

Methods: We evaluated 40 jSLE patients (according to ACR criteria); mean age = 19 ± 4.4 years and mean disease duration = 6 ± 3.2 years; 34 females, 6 males. Twenty healthy children and 20 rheumatic fever patients matched for age and sex were included as controls. All subjects were interviewed in order to search for risk factors for HCV infection including use of endovenous drugs, blood products transfusions, promiscuous sexual activity, previous hospitalizations and/or invasive diagnostic or therapeutic procedures. Serum samples were tested for ANAs by standard techniques and for anti-HCV antibodies using a high sensitive third generation microparticle enzyme immunoassay (AxSYM HCV version 3.0, Abbott Lab.).

## Results

Thirty-six (90%) jSLE patients were under immunossupressive therapy; 47 hospitalizations and 19 invasive procedures were required by jSLE subjects contrasting to only 5 hospitalizations and 5 invasive procedures by the control group. ANA titers were elevated in all 40 jSLE sera and negative in all controls. Remarkably only one (2.5%) jSLE sera was anti-HCV positive compared to all uniformly negative control sera.

## Conclusion

Contrary to adult onset SLE, the low prevalence of anti-HCV antibodies in sera from jSLE patients suggests lack of association of HCV infection and jSLE.

